# Book Notices

**Published:** 1890-01

**Authors:** 


### Book Notices.

THE EAR AND ITS DISEASES; being practical contributions to the study of Otology, by Samuel Sexton, M. D., Aural Surgeon to the New York Eye and Ear Infirmary; Fellow of the American Otological Society; Fellow of the New York Academy of Medicine; Member of the Medical Society of the county of New York, and of the Practitioners' Society of New York. Edited by Christopher J. Colles, M. D. Octavo, 473 pages. Numerous illustrations. Extra muslin, $4.00. New York. William Wood & Company.

From a practical standpoint, this work is not so convenient for reference as many others, though once it has been carefully read, this seeming defect will disappear. For the busy practitioner there is much which is of no practical value, e. g., about 40 pages to othæmatorna, 25 to gun shot, sabre and sword wounds, also much space is given to injures from concussion of large guns, and blast of projectiles in a pleasing and comprehensive style, and must be of great interest to the alienist.

The operation of excision of the drum head and auricles, for otorrhœa and deafness from chronic middle ear catarrh, with a full review of the literature, is worth reading. The conservative course in treating mastoid troubles, comes very opportunely, as the tendency is to great boldness, if not recklessness in opening that cavity. As the author states: "The operation is needlessly resorted to in a great many cases." The book is well worth close study, and those giving it most thought, will like it best.

---

The International Medical Annual and Practitioners' Index. A work of reference for practitioners, or a dictionary of medical progress, by Percy Wilde, M. D., and twenty-five associate editors, European and American. 544 pages, $2.75. E. B. Treat, New York, 1889.

This book intends to give all the new things that happen during the year. It does not include much surgery, but contains a well arranged review of everything medical, in three parts. The first part is devoted to new remedies, the second to new points in respect to treatment, and the third part to miscellaneous information, including a dictionary of new remedies, dictionary of new treatment, etc., arranged somewhat after Bartholow.

The type is rather small, but a worse feature is that abominable Metric system. B.

---

Digest of Criticisms on the United States Pharmacopœia. Sixth Decennial Revision (1880). Published by the Committee of Revision and Publication of the Pharmacopœa of the United States of America (1880-1890). Parts I. and II. New York, 1889.

These books are not for sale. They are advance sheets of the coming pharmacopœia for 1890. These parts are intended to facilitate the final revision of the United States Pharmacopœia, which is to be issued the present year, 1890.

---

A Treatise on Headache and Neuralgia, including spinal irritation and a disquisition on normal and morbid sleep. By J. Leonard Corning, M. D., of New York. pp. 227. Price $2.75. E. B. Treat, New York, 1889.

Dr. Corning is good authority on the subjects treated of in the above book. It is a book that will pay anyone to read and study, though he may be somewhat disappointed at not finding more pathology scattered through its pages. B.

---

A Manual of Diseases of the Ear, for the use of students and practitioners of medicine, by Albert H. Buck, M. D., Clinical Professor of the Diseases of the Ear, in the College of Physicians and Surgeons, New York; Consulting Aural Surgeon, New York Eye and Ear Infirmary. 420 pages. Illustrated. Price, extra muslin, $2.50. New York: William Wood & Company.

This work is intended more for the use of students and general practittioners of medicine than for the specialist. It is very carefully prepared. The main points are admirably emphasized, and made as plain as it is possible to make them. It is a work of real practical value, as might be expected, coming from Dr. Buck. It reflects modern sentiment and research and should be read by every practitioner in the country. B.

---

To Be Justly Reward:—An editor has invented an infernal machine which he places in an envelope and sends to those who "refuse" the paper after taking it five years without paying for it. The machine explodes and kills the whole family and th fragments that fall in the yard kill the dog. Glory certainly awaits that editor, and when he gets into the sanctum that awaits him above he will have an upholstered chair and be allowed to sit with his feet on a tablc.—Porcupine.

